# 

**Published:** 1886-04

**Authors:** 


					﻿i axtfs. a cop}A
APRIL, 1886.
$ i .00 per year.(
HAI
^OVRNAL'J
EIEALT H
ESTABLISHED 1854
W 33'
4
OFFICE, 75 & 77 BARCLAY bTftEET.NEW YORK.
Entered as Second-class Matter at the Post-Office, New York.
				

